# Case report: Unique FLT4 variants associated with differential response to anlotinib in angiosarcoma

**DOI:** 10.3389/fonc.2022.1027696

**Published:** 2022-11-14

**Authors:** Yuanyuan Gu, Jing Meng, Yongzhi Ju, Xia You, Tingting Sun, Jun Lu, Yin Guan

**Affiliations:** ^1^ Department of Medical Oncology, Beijing Chao-Yang Hospital, Capital Medical University, Beijing, China; ^2^ Department of Medical Oncology, The Chinese People’s Liberation Army (PLA) General Hospital, Beijing, China; ^3^ The Medical Department, Jiangsu Simcere Diagnostics Co., Ltd., Nanjing, China; ^4^ Department of Pathology, Beijing Chao-Yang Hospital, Capital Medical University, Beijing, China

**Keywords:** cutaneous angiosarcomas, vascular endothelial growth factor receptor (VEGFR), *FLT4*, anlotinib, soft tissue sarcomas

## Abstract

Angiosarcoma (AS) is a rare, clinically aggressive tumor with limited treatment options and a poor prognosis. Mutations involving the angiogenesis-related genes*TP53*, *PTPRB*, *PLCG1*, *KDR* as well as *FLT4* amplification have been observed in AS. There is a potential therapeutic value of inhibition of the VEGF pathway against angiosarcoma. Our case first described a patient with two sites of cutaneous angiosarcomas (cASs) that responded differently to anlotinib. And genetic analysis revealed that those two sites had different *FLT4* variants, suggesting that *FLT4* amplification could be the cause of anlotinib non-response.

## Introduction

Angiosarcomas (ASs) are rare, aggressive tumors of vascular or lymphatic endothelial cell origin that account for about 2%–3% of all adult soft tissue sarcomas. They can arise from anywhere on the body, with cutaneous angiosarcomas (about 60% of all cases) involving the head and neck, particularly the scalp, followed by the breast, extremity, trunk, liver, and other sites (1, 2). ASs can be divided into primary cutaneous (in the absence of lymphoedema or radiation), parenchymal tissue/visceral (which includes primary breast lesions), deep soft tissue, and lymphoedema-associated and post-radiation angiosarcomas, according to lesion location and underlying risk factors (1, 3). The prognosis of ASs is poor, with approximately 16%–44% of advanced or metastatic patients presenting at the time of diagnosis (2, 4). ASs have a 5-year survival rate of 30%–40% with overall survival ranging from 6 to 16 months depending on tumor stage, surgical resection, and distant metastases (5, 6).

When compared to Western countries, approximately 50%–60% of cutaneous angiosarcomas (cASs) originate in the head and neck, with Asians involving up to 90% of the scalp and face (7). cASs are more common in elderly men between the ages of 60 and 70 who have distinct clinical causative factors, such as chronically sun-damaged skin or chronic lymphedema/irradiated skin (8). Patients with angiosarcoma of the head and neck have a higher tumor mutation burden and a dominant UV damage mutational signature (9, 10).

Surgical resection with wide excisional margins is the main therapy for R0 resection cASs, but due to insufficient surgical excision and the infiltrative growth pattern, local recurrences or metastases are common, particularly in head and neck locations, with 5-year overall survival of 10%–15% (11, 12). Several studies revealed that age, tumor size, tumor location, resection with positive margins, and advanced stage are the poor prognosis factors for cASs (13–15).

Amplifications of *MYC*, *KLT4*, *FLT4*, and recurrent mutations in *TP53*, *PLCG1* (R707), and *PTPRB*, as well as genetic alterations in the mitogen-activated protein kinase (MAPK) pathway, have been reported in ASs (9, 16–19). Many antiangiogenic targeted agents, including vascular endothelial growth factor/receptor inhibitors, such as bevacizumab (20), or multi-kinase inhibitors, pazopanib (21, 22), and sunitinib, have demonstrated clinical efficacy in ASs, including cASs, when used alone or in combination with standard therapies. Clinical success with angiogenesis targeting agents, on the other hand, has so far been lacking.

## Case description

**Figure 1:**
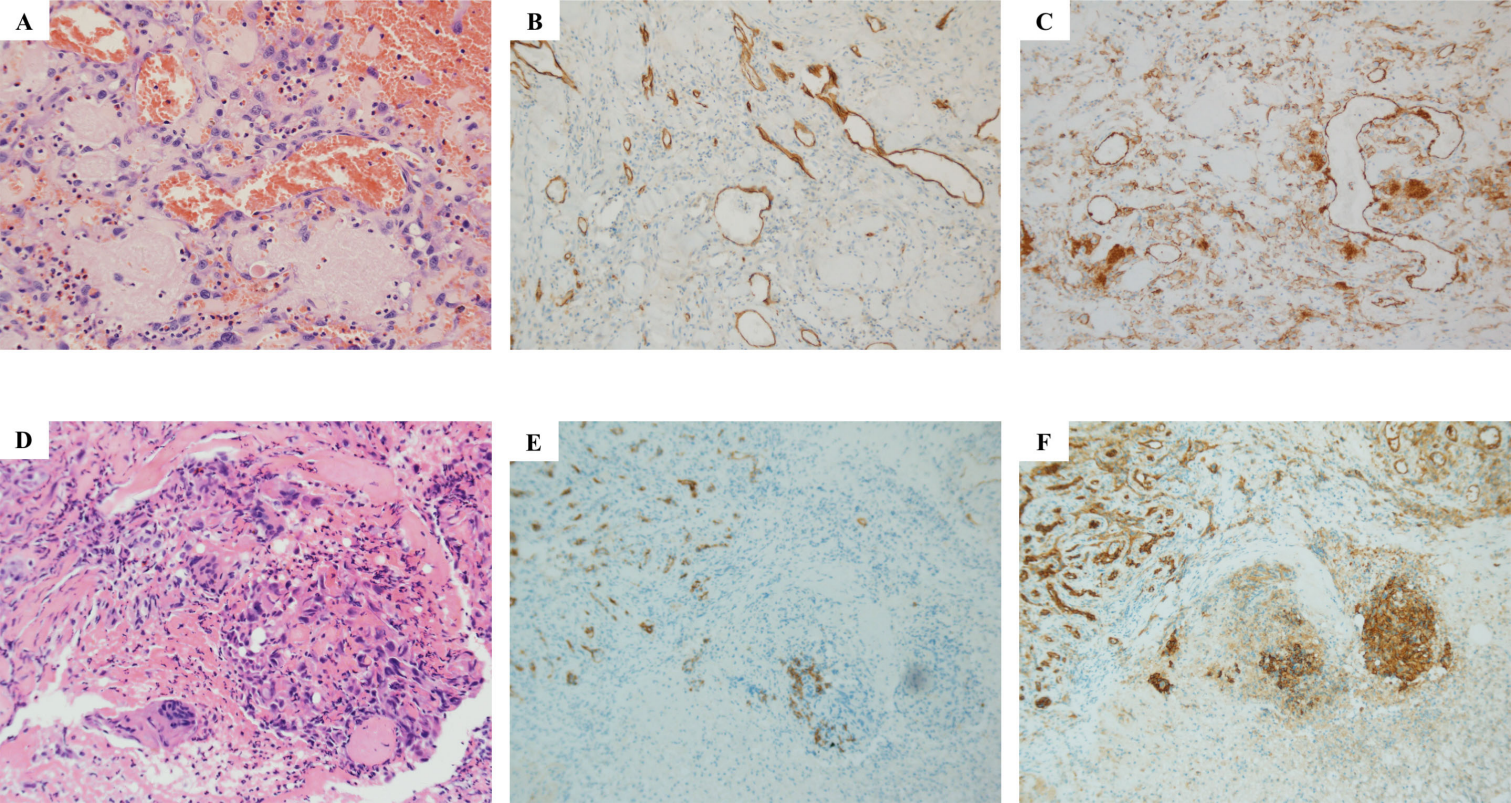
Histopathological examination results of parotid gland and scalp metastasis. Hematoxylin–eosin (HE) staining showed that salivary gland tissue was found in the sample tissue of the left parotid gland mass, and fibrous tissue hyperplasia was observed in some areas **(A–C)** Atypical cells were seen near the granulation tissue, which was indicated by immunophenotype as endovascular cells, and infiltration of lymphocytes and plasma cells was also observed, accompanied by bleeding **(A)**. Immunohistochemical results showed CD34 negative **(B)** and CD31 positive **(C)**. As for the HE result of scalp metastasis **(D, E)**, squamous mucosa, epithelial erosion, a small amount of increased cytoplasmic ratio, irregular karyotype, and trace lesion tissue **(D)** were shown. Immunohistochemical results showed CD34 negative **(E)** and CD31 positive **(F)**.

In December 2018, a 73-year-old male patient discovered on his own a swelling in the left parotid area, initially the size of peanut rice, with local pressure pain but no fever, facial palsy, or taste disturbance. One month later, the swelling had grown to the size of a small date and was diagnosed as a “parotid cyst” at an outside hospital and underwent “left parotid abscess excision.” In March 2019, the patient visited our hospital for a reexamination. The CT scan revealed an irregular mass in the posterior and inferior portion of the left parotid gland, measuring 3.7 × 3.4 cm. This mass had uneven density, mild to moderate enhancement, uneven edges, a poor boundary with the parotid gland, and local skin thickening. Pathological findings postoperatively showed that vascular endothelial derived cells were responsible for the infection, and the immunohistochemical staining showed that CD31 was diffusely positive and the Ki-67 proliferation index was 50%–60%, whereas CD34 was negative (**Figures 1A–C**), with lymphoid and plasma cell infiltration and bleeding. The presence of two lymph nodes surrounding the tissue led to a preliminary diagnosis of poorly differentiated AS.

**Figure 2:**
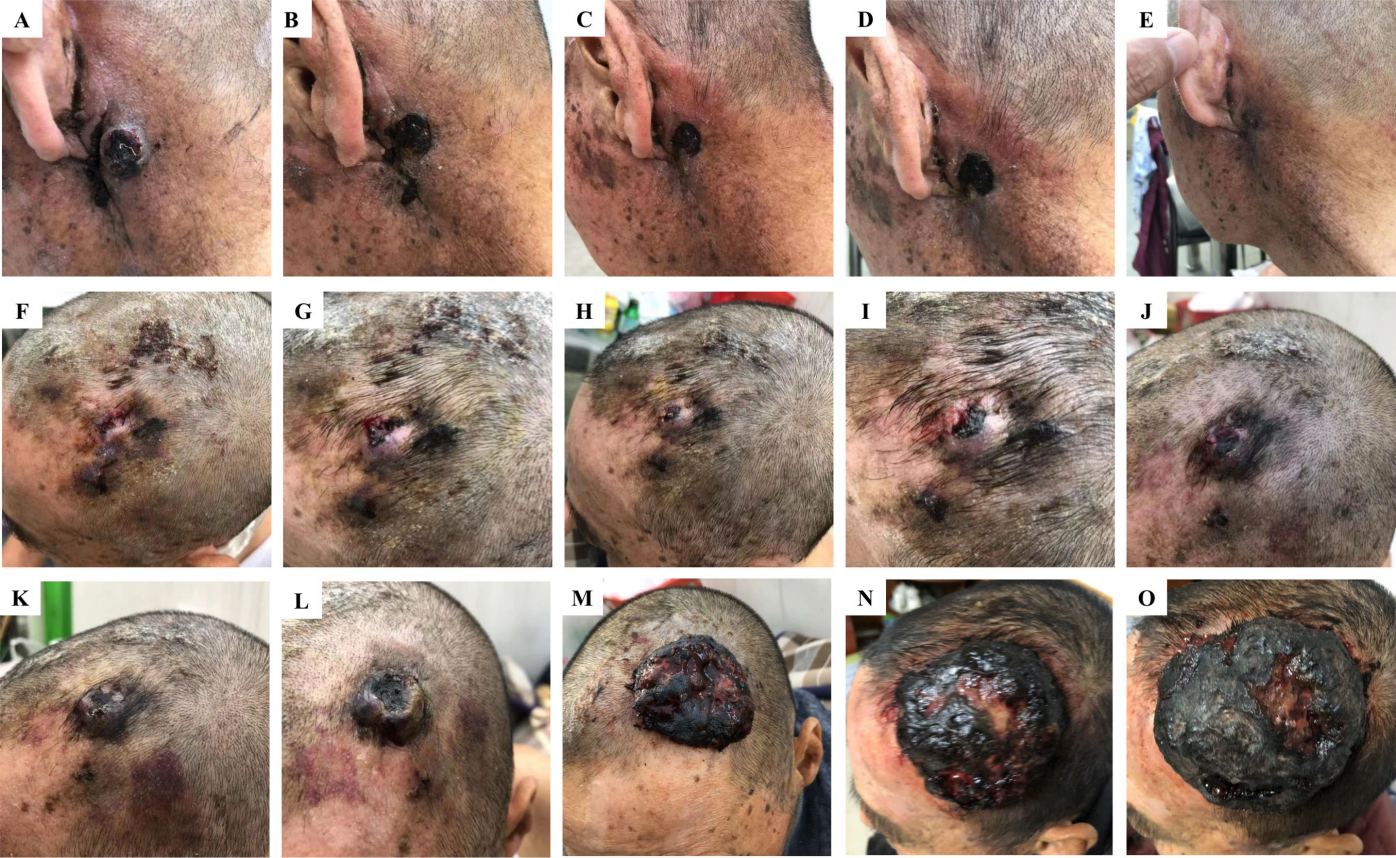
Different responses of retro auricular and scalp angiosarcoma metastases to anlotinib treatment. Changes in retro auricular recurrence and overhead metastases during anlotinib treatment on 13 July, 22 July, 25 July, 28 July, and 1 August 2019, respectively. Among them, the retro auricular tumor was relieved quickly after anlotinib treatment, and the bump was gradually reduced to disappear, achieving recovery **(A–E)**. However, the scalp metastasis of the same period was not effective, and the lesion gradually deteriorated until it was uncontrollable **(F–O)**. Continuous deterioration of the scalp metastatic during anlotinib maintenance on 4 August **(K)**, 12 August **(L)**, 8 October **(M)**, 19 November **(N)**, and 10 December 2019 **(O)**.

However, a relapse of the local bump behind the left ear 2 months after the operation. Meanwhile, a lesion appeared on the scalp. The scalp was broken locally, the wound was poorly healed, and the surrounding tissues were eroded. A local biopsy revealed CD31, ERG, and Ki-67 were positive, P53 was partially positive, while CK, CD34, CD117, CEA, and P40 were negative, indicating that this was a metastasis (**Figures 1D–F**). Given the size of the lesion, the patient’s old age, and physical condition, surgery and chemotherapy were risky treatments, and the patient made an informed decision to decline them. So anlotinib, a multi-targeted tyrosine kinase inhibitor approved as second-line treatment for advanced soft-tissue sarcoma, was recommended after complete informed consent in July 2019. During the treatment, the tumor behind the left ear was quickly relieved until it disappeared **(Figures 2A–E)**, but anlotinib was not effective for scalp metastasis, and the lesions continued to deteriorate until they were uncontrollable (**Figures 2F–O**). This patient died in May 2020 without a relapse behind the left ear.

There was a significant difference in the response to anlotinib between recurrent parotid lesions and scalp metastases. We sequenced the scalp metastatic and the surgically resected primary tissues by Next Generation Sequencing (NGS). The sequencing results showed that the primary lesions only had the *FLT4* p.G1276E mutation. However, in addition to the *FLT4* p.G1276E mutation, scalp metastasis also exhibited *FLT4* amplification and mutations of *FLT1/ARID1A/CHEK2/IRS1* (**Table 1**).

**Table 1:** DNA-NGS detection results of the patient.

Genes	Primary tumor	Scalp metastasis
*FLT4*	p.Gly1276Glu (25.58%)	p.Gly1276Glu (68.23%)
*FLT4*		Amplification (CN: 4.9)
*FLT1*		p.Gly181Glu (4.86%)
*ARID1A*		p.Gln425* (3.25%)
*CHEK2*		p.Arg3Trp (3.72%)
*IRS1*		p.Thr1103Ala (2.91%)
*TMB*	0.8 Muts/Mb	4.0 Muts/Mb
*MSI*	MSS	MSS

muts/Mb, mutations per megabase; NGS, next-generation sequencing; TMB, tumor mutational burden; MSI, microsatellite Instability; CN, Copy number.

Identification of genomic alterations to confirm predictive biomarkers for anti-angiogenesis therapy is always on the way. The predictive value of the *FLT4* F131S mutation to regorafenib was not ruled out because of its extracellular location and complicated genomic profiles (23). Although a 6-month clinical stable response to pazopanib caught our attention in *VEGFR*-2 or 3 amplified angiosarcoma (24), we should not ignore the potential responsive mechanism of the *VEGFR3* R1070L mutation in the tyrosine kinase domain. In our description, the unique and pure *FLT4* G1276E mutation may be a reasonable explanation for this rapid and durable response to anlotinib. The amplification of *FLT4* by metastatic lesions may be the reason for the unresponsiveness after constructional analysis of other gene alterations. Further study is ongoing and warranted.

## Conclusion

AS is a rare, highly heterogeneous sarcoma with a variety of clinical characteristics. The prognosis of ASs is poor, with a 5-year survival rate of only 30%–40% from primary diagnosis, but once metastasized, the average survival period is less than 1 year. Based on the stage and the location, radical surgery remains the curative treatment for Ass, and adjuvant radiochemotherapy is mainly used to treat locally advanced or progressive patients (6). Targeted agents, including mono-target antibodies, bevacizumab, or multi-target small molecules in tyrosine kinases, pazopanib, and regorafenib, have shown significant clinical efficacy (25). There are some immunotherapy drugs, and combination strategies are being investigated in various subtypes of soft tissue sarcomas, including ASs (26). Due to the complex correlation between drug efficiency and pathological subtype heterogeneity as well as genetic characteristics, treatment options for ASs remain limited (27).

Anlotinib is a new oral molecular multi-target tyrosine kinase inhibitor (TKI) that targets vascular endothelial growth factor receptor (*VEGFR*) 1, *VEGFR3*, *VEGFR2*/KDR, platelet-derived growth factor receptor a (*PDGFR*-a), c-Kit, and fibroblast growth factor receptor (FGFR) 1–3 and inhibits tumor angiogenesis and tumor cell proliferation. It has been approved as a third-line treatment for patients with advanced non-small-cell lung cancer (NSCLC) (28). Based on a preclinical study, anlotinib showed high selectivity for VEGF family members, especially VEGFR2 and VEGFR3, with IC50 values of 0.2 and 0.7 nmol/L, showing high selectivity and inhibitory potency to sunitinib (29). A phase 2 clinical trial involving 166 patients to evaluate anlotinib in recurrent metastatic soft tissue sarcomas found that the overall 12-week PFS and ORR rates were 68% and 13%, respectively, with easily controllable adverse events (30). More trials are being conducted to investigate the efficacy of anlotinib monotherapy, anlotinib plus immunotherapy, and anlotinib plus chemotherapy in advanced sarcomas, particularly soft tissue sarcomas (31). So far, there have been few reports on the efficacy of anlotinib treatment in ASs, with only a few case reports referring to various lesions in head and neck AS (32) and cardiac AS (33) or combined immunotherapy in metastatic primary splenic AS (34). In the case we present, the patient experienced local recurrence in the ear as well as scalp metastases following surgery. Anlotinib was an option chosen when the patient declined chemotherapy and surgery. However, the two lesions respond differently to treatment, with the lesion behind the ear disappearing quickly and the lesion on the scalp worsening. *FLT4* p.G1276E mutation rate doubled, and newly added *FLT4* amplification as well as point mutation of *FLT1/ARID1A/CHEK2/IRS1*, according to genetic alterations from primary and metastatic tissues.

The *FLT4* gene encodes VEGFR3 and is involved in lymphatic differentiation. It has been reported that inappropriate genetic abnormalities of VEGFR signaling are critical in the genesis of angiosarcomas (35, 36). *FLT4* gene amplification, along with *MYC*, is present in approximately 25% of secondary angiosarcomas (17). A case report described a 6-month clinically stable response to pazopanib in an angiosarcoma patient with *VEGFR*-2, *VEGFR*-3 amplification, and a novel *VEGFR3* R1070L mutation (24). Arnaud-Coffin et al. (37) also reported a partial response to pazopanib in an *FLT4* amplified angiosarcoma patient, but with a worse PFS of 3.1 months. *FLT* 4 amplification was also reported to be a poor prognostic factor for ASs (38). Several basket trials with *FLT4* gene amplification subgroups are also being conducted (NCT02693535, NCT02029001, NCT03297606). The *FLT1* gene encodes VEGFR1 and is a member of the VEGFR family. The point mutation c.542G>A was found in *FLT1*’s Ig-like domain 2, which is a high-affinity ligand-binding region (29). Some studies have found that soluble *FLT1* expression is associated with poor clinical outcomes and may be a biomarker of intrinsic resistance to anti-angiogenic therapies that selectively inhibit the VEGFR-2 signaling pathway (39). However, no studies have been conducted to investigate the impact of *FLT1* extracellular domain mutations on the efficacy of kinase inhibitor therapy.

*ARID1A* encodes BAF250A, which is a member of the SWI/SNF chromatin-remodeling complex. *ARID1A* is a tumor suppressor, and most mutations seen in cancers are frameshift or nonsense mutations that result in inactivation and reduced expression of *ARID1A* (40). The overall mutation rate of *ARID1A* in cancers is about 6%, with clear-cell ovarian cancer (45%) and endometrial cancer (37%) being the most common (41, 42). There is a lot of evidence that *ARID1A* loss-of-function mutations can activate the PI3K/Akt/mTORpathway (43, 44) and increase the sensitivity of ARID1A-deficient cells to treatment with the PI3K/AKT inhibitor in gastric cancer cells (45) or cholangiocarcinoma (46). Checkpoint kinase 2 (CHEK2) is a cancer susceptibility gene that codes for the serine/threonine CHK2 kinase involved in DNA damage response (DDR). A higher frequency of germline mutations is significantly associated with breast and ovarian cancers (47). Insulin receptor substrate-1 (IRS1) is a mediator of oncogenic IGF signaling and is overexpressed in a variety of malignant tumor types, where it also mediates EGFR or mTOR inhibitor resistance (48–50). Although these mutations are linked to sarcoma development to some extent, there have been no reports of these mutations being linked to sarcoma-targeted therapy resistance.

In our case, *FLT4* p.G1276E is a novel mutation localized in the carboxyterminal domain of *VEGFR3* that may be the primary reason for the response to anlotinib in the recurrence site, but the *FLT4* amplification in the metastatic site may be a worse prognosis factor and showed no response to anlotinib. Our findings may broaden the spectrum of the *FLT4* gene mutations while also providing guidance for clinical anlotinib treatment of local recurrence and metastatic ASs.

## Data availability statement

The original contributions presented in the study are included in the article/supplementary material. Further inquiries can be directed to the corresponding author.

## Ethics statement

Written informed consent was obtained from the individual(s) for the publication of any potentially identifiable images or data included in this article.

## Author contributions

YiG treated and observed the patient. YuG, JM, and YiG collected the clinical information, diagnostic information, therapeutic information, and images of the patients. JL performed the histopathological and immunohistochemical examinations. YiG, YuG, JM, and JL prepared the manuscript and the literature search. YJ, XY, and TS performed data analysis and interpretation. YiG and JM reviewed and edited the manuscript. All authors contributed to the article and approved the submitted version.

## Conflict of interest

Authors YJ, XY, and TS are employed by Jiangsu Simcere Diagnostics Co., Ltd.

The remaining authors declare that the research was conducted in the absence of any commercial or financial relationships that could be construed as a potential conflict of interest.

## Publisher’s note

All claims expressed in this article are solely those of the authors and do not necessarily represent those of their affiliated organizations, or those of the publisher, the editors and the reviewers. Any product that may be evaluated in this article, or claim that may be made by its manufacturer, is not guaranteed or endorsed by the publisher.
